# The Impact of Frailty on VARC-3 Integrated Outcomes in Patients Undergoing Transcatheter Aortic Valve Replacement

**DOI:** 10.1016/j.jacadv.2025.101594

**Published:** 2025-02-14

**Authors:** Daijiro Tomii, Jonas Lanz, Dik Heg, Helge Möllmann, Won-Keun Kim, Christof Burgdorf, Axel Linke, Simon Redwood, Michael Hilker, Michael Joner, Holger Thiele, Lenard Conradi, Sebastian Kerber, Christian Thilo, Stefan Toggweiler, Bernard Prendergast, Thomas Walther, Stephan Windecker, Thomas Pilgrim, SCOPE I Investigators

**Affiliations:** a Department of Cardiology, Bern University Hospital, Inselspital, University of Bern, Bern, Switzerland; b Department of Clinical Research, University of Bern, Bern, Switzerland; c Department of Internal Medicine I, St-Johannes-Hospital, Dortmund, Germany; d Department of Cardiology, Kerckhoff Heart and Thorax Centre, Bad Nauheim, Germany; e Department of Cardiology, Heart, and Vascular Center, Bad Bevensen, Germany; f Department of Internal Medicine and Cardiology, Heart Centre Dresden, Technische Universität Dresden, Dresden, Germany; g Department of Cardiology, St Thomas’ Hospital & Cleveland Clinic, London, United Kingdom; h Department of Cardiothoracic Surgery, University Medical Centre, Regensburg, Germany; i German Heart Centre, Technical University of Munich, Munich, Germany; j Department of Cardiology, Heart Center Leipzig, Leipzig, Germany; k Department of Cardiovascular Surgery, University Heart Centre Hamburg, Hamburg, Germany; l Department of Cardiology, Cardiovascular Centre Bad Neustadt, Bad Neustadt, Germany; m Department of Internal Medicine I, RoMed Klinikum, Rosenheim, Germany; n Heart Center Lucerne, Luzerner Kantonsspital, Lucerne, Switzerland; o Department of Cardiac, Thoracic and Thoracic Vascular Surgery, University Hospital Frankfurt, Frankfurt, Germany

**Keywords:** aortic stenosis, frailty, transcatheter aortic valve replacement, valve academic research consortium

## Abstract

**Background:**

TAVR is preferred over surgical aortic valve replacement in frail patients with aortic stenosis. The assessment of the treatment benefit of TAVR in this population is however equivocal.

**Objectives:**

The purpose of this study was to investigate the impact of frailty on clinical and patient-reported outcomes in patients undergoing transcatheter aortic valve replacement (TAVR).

**Methods:**

Patients in the SCOPE I (Safety and Efficacy of the ACURATE Neo/TF Compared to the SAPIEN 3 Bioprosthesis) trial were stratified according to frailty, defined as a multicomponent index that included loss of independence criteria based on activities of daily living, lean body mass, serum albumin, and cognitive impairment or dementia. The outcomes of interest included an endpoint integrating vital and patient-reported disease-specific health status, as well as clinical efficacy according to the Valve Academic Research Consortium (VARC)-3 definition.

**Results:**

Among 739 randomized patients, 122 patients (16.5%) met the definition of frailty. Mean age, comorbidities, and surgical risk were comparable between groups. Patients with and without frailty had similar improvement in patient-reported health status measures after TAVR, while patients with frailty had an increased risk of VARC-3 unfavorable outcome (risk ratio: 1.38, 95% CI: towards reduced VARC-3 clinical efficacy (risk ratio: 0.82; 95% CI: 0.65-1.03) at 3 years after TAVR.

**Conclusions:**

More than 1 in 6 patients with severe aortic stenosis undergoing TAVR were considered frail in the SCOPE I trial. Patients with frailty had a similar improvement in patient-reported health status measures after TAVR, but a higher risk of unfavorable outcomes throughout 3 years of follow-up.

As a consequence of the demographic transition, a substantial proportion of patients with cardiovascular disease have significant comorbidities, which render treatment decisions more complex and interventions more challenging.^1^ Frailty is a multidimensional geriatric syndrome defined as slowness, weakness, exhaustion, wasting and malnutrition, poor endurance and inactivity, and loss of independence.^2^ An important proportion of patients with severe degenerative aortic stenosis (AS) have coexisting frailty when referred for aortic valve replacement (AVR).^3^^,^^4^ Transcatheter aortic valve replacement (TAVR) has consistently been shown to prolong survival and improve quality of life,^5^, ^6^, ^7^ Frailty is not directly affected by TAVR and has been associated with poor clinical outcomes and reduced quality of life even after AVR.^3^^,^^4^^,^^8^, ^9^, ^10^, ^11^, ^12^, ^13^, ^14^, ^15^, ^16^, ^17^, ^18^ As a consequence, it remains to be determined whether the favorable effect of TAVR in patients with frailty is limited to survival benefit while expanding morbidity, rather than improving patient-reported outcome measures. Evaluation of treatment success is therefore particularly challenging in this patient population. Available evidence describes clinical outcomes and health status as a composite outcome, which complicates interpretation of the benefit and futility of AVR on disease-specific health status in this population.^19^ Given that survival alone is unlikely to fully encompass the goals of disease management (especially in an older population) and that disease-specific health status may improve over time due to attrition of the sickest patients, integration of clinical and patient-reported quality of life measures is likely to be more appropriate to provide a comprehensive assessment of the effect of AVR in frail patients with severe AS. The current Valve Academic Research Consortium (VARC)-3 outcome criteria propose composite endpoints that integrate clinical outcomes and patient-reported health status measures.^19^^,^^20^ In the present study, we aimed to investigate an integrated endpoint of clinical outcomes and health-related quality of life measures according to the presence of frailty in patients undergoing TAVR.

## Methods

### Study design and population

SCOPE I (Safety and Efficacy of the ACURATE Neo/TF Compared to the SAPIEN 3 Bioprosthesis) was an investigator-initiated, randomized clinical trial conducted at 20 tertiary heart centers across Europe comparing the self-expanding Acurate Neo (Boston Scientific) to the balloon-expandable SAPIEN 3 (Edwards Lifesciences) transcatheter heart valve (THV) in older patients with symptomatic severe AS undergoing TAVR. Details of the design and conduct of the trial have been previously reported.^21^^,^^22^ In brief, patients aged 75 years or older with symptomatic, severe AS who were deemed to be at increased surgical risk by the heart team were randomized in a 1:1 ratio to undergo transfemoral TAVR with an Acurate Neo or SAPIEN 3 THV. Key exclusion criteria included pre-existing left-sided prosthetic valves, need for an emergency procedure, severely reduced left ventricular ejection fraction (<20%), any concomitant procedure except for a percutaneous coronary intervention, stroke or myocardial infarction within 30 days before valve implantation, and any planned noncardiac surgery within 30 days after implantation. The trial was approved by an appropriately constituted institutional review board at each site and conducted in accordance with the Declaration of Helsinki (NCT03011346).

### Frailty assessment

Frailty was evaluated during the screening process and incorporated into the Heart Team’s assessment and recommended treatment strategy (TAVR or surgical aortic valve replacement, SAVR). To provide a multidimensional framework that captures the impact of frailty across physical, cognitive, nutritional, and endurance dimensions—particularly relevant to an aging, highly comorbid TAVR population—frailty was defined as a biological syndrome characterized by decreased reserve and resistance to stressors, resulting from cumulative declines across multiple physiological systems, including slowness, weakness, exhaustion, reduced walking performance and balance, age-related decline in lean body mass, wasting, malnutrition, poor endurance, and inactivity in a broad sense.^2^ An electronic case report form was used to collect the index as multiple components encompassing loss of independence criteria: extended time to walk 5 m, reduced grip strength, body mass index <20 kg/m^2^ and/or weight loss 5 kg/y, serum albumin <35 g/L, and cognitive impairment or dementia.

### Data collection and clinical endpoints

In the trial, regular follow-up was performed at 30 days and at 1 and 3 years by means of outpatient visits, telephone interviews and consultation of medical records. Clinical endpoints were adjudicated by an independent clinical event committee blinded to treatment allocation according to VARC-2 criteria (Cardiovascular European Research Center, Massy, France).^21^^,^^23^ Patient-reported health status was assessed using the Kansas City Cardiomyopathy Questionnaire (KCCQ)-12 score.^21^^,^^22^^,^^24^^,^^25^ The KCCQ encompasses four domains: physical limitation, quality of life, social limitation, and symptom frequency, and these domains were combined into an overall summary score. For the purpose of the present study, an ordinal analysis of overall KCCQ score was applied based on changes from baseline:^20^^,^^26^ severe decrease (decrease by >10 points); decrease (decrease by 5-10 points); no change (change between −5 and 5 points); mild improvement (increase by 5-10 points); moderate improvement (increase by 10-20 points); substantial improvement (increase ≥20 points). The VARC-3 definitions recommend reporting both the integrated categorical endpoint of clinical outcomes and disease-specific health status as well as patient-reported health status, especially in cases where there is a survival difference between groups. According to the recommendation, we retrospectively adjudicated the integrated endpoints (including general outcomes and clinical efficacy) based on the adjudicated clinical endpoints and overall KCCQ score at 1 and 3 years of follow-up:^20^

• Favorable outcome: a patient is alive, AND has an overall KCCQ score ≥60, AND has not had a decline of >10 points in overall KCCQ score from baseline;
• Acceptable outcome: a patient is alive, AND has an overall KCCQ score ≥45, AND has not had a decline of >10 points in overall KCCQ score from baseline;
• Unfavorable outcome: a patient is not alive OR is alive AND has either an overall KCCQ score <45 OR has had a decline of >10 points in overall KCCQ score from baseline; and
• Clinical efficacy: Freedom from all-cause mortality AND freedom from all stroke AND freedom from hospitalization for procedure- or valve-related causes AND freedom from unfavorable outcome.

### Statistical analysis

Categorical variables are presented as frequencies and percentages, and differences between groups were evaluated with the chi-square test or Fisher exact test in case of 2 × 2 comparisons. Continuous variables are presented as mean ± SD and compared using the Student’s *t*-test, given that the central limit theorem supports the assumption of normality in large samples. To verify this, Shapiro-Wilk tests and Q-Q plots were conducted, flagging some variables as potentially non-normally distributed. Recognizing the sensitivity of these tests in large samples, we included medians and interquartile ranges in Table 1 and used Mann-Whitney *U* tests for robustness, confirming consistent findings. The Hodges-Lehmann median difference was calculated to estimate the difference in KCCQ scores between patients with and without frailty and groups compared using the Mann-Whitney. Alive patients without information on KCCQ score at baseline or follow-up were not included in the analysis of clinical efficacy (n = 106 and 148 at 1 and 3 years, respectively), whereas deceased patients remained in the denominator throughout. To address missing variables, a sensitivity analysis was performed using multiple imputation with chained equations. Predictive mean matching was used for continuous variables, and 5-nearest-neighbor kernel matching was applied for categorical data. Proportions with 95% CI and risk ratios (RRs) with 95% CIs were estimated from 20 imputed datasets using Rubin’s rule. Cumulative incidence curves were constructed using the Kaplan-Meier method for time-to-event data (death, stroke, and hospitalization for valve-related dysfunction or heart failure; counting only the first occurrence per patient for the latter two event categories), censoring patients at last valid contact (or after death in those who were deceased); 8 patients withdrew consent before 30 days and no clinical outcomes were recorded (frail, n = 2; nonfrail, n = 6). Cox proportional hazards models were used to calculate HR and 95% CIs for time-to-event outcomes, and RR with 95% CIs from Poisson regressions were provided for VARC-3 integrated endpoint. It was anticipated that traditional Cox proportional hazards models may overestimate event rates when competing with death in this elderly population with relevant comorbidities. To account for this limitation, we performed a competing risk analysis using the Fine and Gray method to model the cumulative incidence function for the outcomes of interest (cardiovascular death, all stroke, and hospitalization for valve-related dysfunction or heart failure) and to determine the subdistribution HR under competing risk of death or, in the case of cardiovascular death, under competing risk of noncardiovascular death.^27^, ^28^, ^29^ Furthermore, we investigated the relationship between frailty, THV type, and outcomes using a full factorial design that allows the main effects of factors and their interactions to be examined simultaneously while controlling for type I errors.^30^^,^^31^ All statistical tests were 2-sided and *P* values <0.05 were considered statistically significant. Statistical analyses were performed using Stata 18 (StataCorp).

**Table 1:** Baseline and Procedural Characteristics According to Frailty

	All Patients (N = 739)	Patients With Frailty (n = 122)	Patients Without Frailty (n = 617)	Difference (95% CI)	*P* Value
Age, y	83 ± 4	82 ± 4	83 ± 4	−0.63 (−1.43 to 0.17)	0.12
Age, y	83.0 (80.0, 85.0)	83.0 (80.0, 85.0)	83.0 (80.0, 86.0)	-	0.20
Female	420 (56.8%)	82 (67.2%)	338 (54.8%)	−12% (−22% to −3%)	0.012
Body mass index, kg/m^2^	27.6 ± 4.6	27.8 ± 4.6	27.6 ± 4.5	0.23 (−0.65 to 1.12)	0.61
Body mass index, kg/m^2^	27.0 (24.2, 30.7)	27.4 (24.6, 30.8)	26.9 (24.2, 30.6)	-	0.40
Body mass index <20 kg	16 (2.1%)	6 (4.9%)	10 (1.6%)	3% (0%-6%)	0.035
STS-PROM, %	4.3 ± 2.9	4.70 ± 4.2	4.2 ± 2.5	0.48 (−0.08 to 1.04)	0.090
STS-PROM, %	3.5 (2.6, 5.0)	3.6 (2.8, 5.3)	3.5 (2.5, 5.0)	-	0.16
NYHA III or IV	554 (75.0%)	104 (85.2%)	450 (72.9%)	12% (4%-21%)	0.004
CCS grade III or IV	44 (6.0%)	6 (4.9%)	38 (6.2%)	−1% (−6% to 3%)	0.83
Syncope (%)	81 (11.0%)	15 (12.3%)	66 (10.7%)	2% (−4% to 8%)	0.63
Low activity	265 (38.8%)	60 (52.6%)	205 (36.0%)	17% (7%-26%)	0.001
Dementia/cognitive impairment	14 (1.9%)	4 (3.3%)	10 (1.6%)	2% (−1% to 4%)	0.27
Serum albumin <35 g/L	132/543 (24.3%)	14/94 (14.9%)	118/449 (26.3%)	−11% (−21% to −2%)	0.018
Serum albumin, g/L	38.9 ± 5.6	40.3 ± 5.0	38.6 ± 5.7	1.71 (0.47-2.96)	0.007
Serum albumin, g/L	39.0 (35.0, 43.0)	40.6 (37.5, 43.7)	38.4 (34.0, 43.0)	-	0.010
Comorbidities					
Hypertension	674 (91.2%)	112 (91.8%)	562 (91.1%)	1% (−5% to 6%)	1.00
Diabetes mellitus	224 (30.3%)	35 (28.7%)	189 (30.6%)	−2% (−11% to 7%)	0.75
Creatinine concentration >2 mg/dL	28 (3.8%)	4 (3.3%)	24 (3.9%)	−1% (−4% to 3%)	1.00
Coronary artery disease	437 (59.1%)	69 (56.6%)	368 (59.6%)	−3% (−13% to 6%)	0.55
Previous myocardial infarction	86 (11.6%)	18 (14.8%)	68 (11.0%)	4% (−3% to 10%)	0.28
Previous stroke or TIA	94 (12.7%)	22 (18.0%)	72 (11.7%)	6% (−0% to 13%)	0.073
COPD	77 (10.4%)	15 (12.3%)	62 (10.0%)	2% (−4% to 8%)	0.52
Atrial fibrillation	271 (36.7%)	50 (41.0%)	221 (35.8%)	5% (−4% to 15%)	0.30
Extracardiac arteriopathy	86 (11.6%)	13 (10.7%)	73 (11.8%)	−1% (−7% to 5%)	0.88
Echocardiography^a^					
Aortic valve area, cm^2^	0.73 ± 0.19	0.70 ± 0.19	0.73 ± 0.19	−0.03 (−0.06 to 0.01)	0.16
Aortic valve area, cm^2^	0.7 (0.6, 0.9)	0.7 (0.6, 0.8)	0.7 (0.6, 0.9)	-	0.14
Mean aortic valve gradient, mm Hg	42.2 ± 16.2	42.9 ± 17.6	42.0 ± 15.9	0.86 (−2.30 to 4.01)	0.59
Mean aortic valve gradient, mm Hg	41.0 (31.0, 51.0)	42.0 (30.8, 49.2)	41.0 (31.0, 51.0)	-	0.73
Left ventricular ejection fraction, %	56.7 ± 10.9	55.4 ± 10.9	57.0 ± 10.9	−1.57 (−3.69 to 0.55)	0.15
Left ventricular ejection fraction, %	60.0 (53.0, 65.0)	60.0 (51.0, 60.0)	60.0 (54.0, 65.0)	-	0.036
Moderate or severe aortic regurgitation	78 (11.4%)	15 (13.9%)	63 (10.9%)	3% (−4% to 10%)	0.41
Moderate or severe mitral regurgitation	111 (15.1%)	18 (14.8%)	93 (15.1%)	−0% (−7% to 7%)	1.00
Moderate or severe tricuspid regurgitation	82 (11.5%)	13 (10.8%)	69 (11.6%)	−1% (−7% to 6%)	1.00
Procedural characteristics					
Transfemoral TAVI not initiated	7 (0.9%)	2 (1.6%)	5 (0.8%)	1% (−1% to 3%)	0.33
Transfemoral access mode	N = 732	n = 120	n = 612		0.38
Percutaneous	728 (99.5%)	120 (100%)	608 (99.3%)	1% (−1% to 2%)	1.00
Surgical cut-down	4 (0.5%)	0	4 (0.7%)	−1% (−2% to 1%)	1.00
General anesthesia	160 (21.9%)	14 (11.7%)	146 (23.9%)	−12% (−20% to −4%)	0.002
Valve type implanted	N = 732	N = 120	N = 612		0.14
SAPIEN 3	368 (50.3%)	53 (44.2%)	315 (51.5%)	−7% (−17% to 2%)	0.16
Acurate Neo	364 (49.7%)	67 (55.8%)	297 (48.5%)	7% (−2% to 17%)	0.16
Valve size implanted, mm	25.1 ± 1.6	25.2 ± 1.5	25.1 ± 1.6	0.07 (−0.24 to 0.39)	0.66
Valve size implanted, mm	25.0 (23.0 to 26.0)	25.5 (23.0, 26.0)	25.0 (23.0, 26.0)	-	0.48
Moderate or severe paravalvular regurgitation	27 (3.8%)	3 (2.6%)	24 (4.0%)	−1% (−5% to 2%)	0.60
Procedural complications within 30 days	N = 731	N = 120	N = 611		
Valve malposition	7 (1.0%)	1 (0.8%)	6 (1.0%)	−0% (−2% to 2%)	1.00
Coronary artery obstruction	1 (0.1%)	0	1 (0.2%)	−0% (−1% to 0%)	1.00
Myocardial infarction	4 (0.5%)	1 (0.8%)	3 (0.5%)	0% (.% to .%)	0.51
Implantation of multiple valves	13 (1.8%)	3 (2.5%)	10 (1.6%)	1% (−2% to 3%)	0.46
Cardiac tamponade	9 (1.2%)	1 (0.8%)	8 (1.3%)	−0% (−3% to 2%)	1.00
Annular rupture	1 (0.1%)	0	1 (0.2%)	−0% (−1% to 0%)	1.00
Left ventricular perforation	1 (0.1%)	0	1 (0.2%)	−0% (−1% to 0%)	1.00
Conversion to open heart surgery	3 (0.4%)	0	3 (0.5%)	−0% (−2% to 1%)	1.00
Immediate procedural death	4 (0.5%)	1 (0.8%)	3 (0.5%)	0% (.% to .%)	0.51

Values are mean ± SD, median (Q1, Q3), or n (%) unless otherwise indicated.

CCS = Canadian Cardiologists Society; COPD = chronic obstructive pulmonary disease; NYHA = New York Heart Association; STS-PROM = Society of Thoracic Surgeons Predicted Risk of Mortality: TAVI = transcatheter aortic valve implantation; TIA: transient ischemic attack.

a Transthoracic echocardiography used, if not available transesophageal echocardiography used; if no echocardiography available, cardiac catheterization data used.

## Results

### Study population and prevalence of frailty

A total of 739 patients (Acurate Neo, n = 372; SAPIEN 3, n = 367) were included in the SCOPE I trial between February 2017 and February 2019. Mean age of the study population was 82.8 ± 4.1 years, with 420 (56.8%) female and a mean Society of Thoracic Surgeons Predicted Risk of Mortality (STS-PROM) score of 4.3% ± 2.9%. Prespecified criteria for frailty were met by 122 patients (16.5%) (Table 1), who were more likely to be female (67.2% vs 54.8%, *P* = 0.012) with a higher baseline prevalence of advanced heart failure symptoms (NYHA functional class III or IV: 85.2% vs 72.9%; *P* = 0.004). Mean age, prevalence of comorbidities, and STS-PROM were comparable between groups (Central Illustration), as well as severity of AS, left ventricular ejection fraction, and the prevalence of concomitant moderate or severe valvular heart disease. Procedural characteristics and complications stratified according to the presence or absence of frailty are shown in Table 1. A procedure was not initiated in 7 patients (frail, n = 2; nonfrail, n = 5: *P* = 0.33) and frail patients were less likely to undergo TAVR under general anesthesia (11.7% vs 23.9%, *P* = 0.002). There were no differences in the distribution of the type or size of THV, or the frequency of procedural complications.

**Central Illustration:**
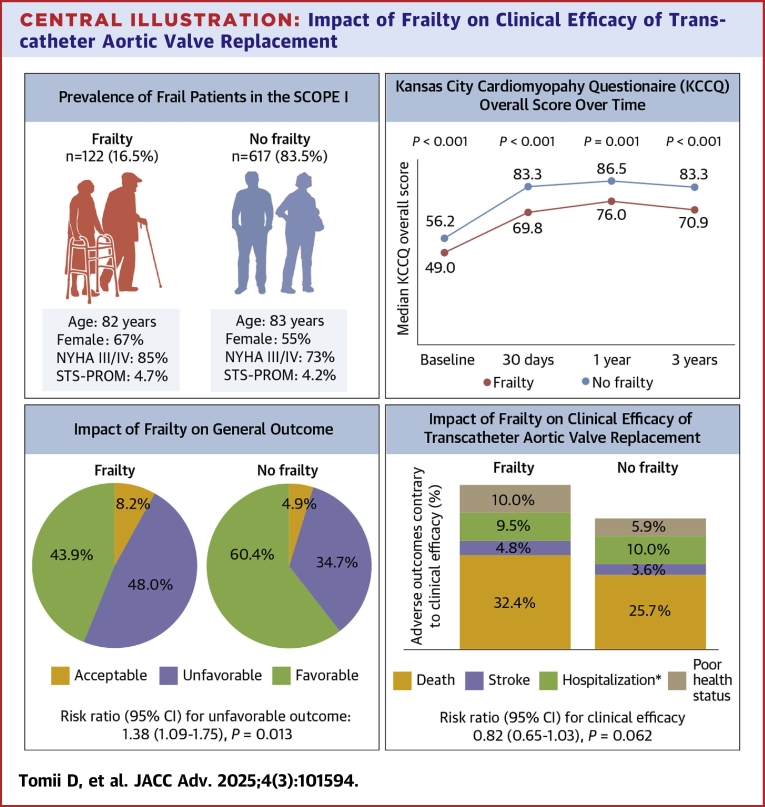
**Impact of Frailty on Clinical Efficacy of Transcatheter Aortic Valve Replacement** Patient demographics at baseline (upper left), overall KCCQ score over the study period (upper right), general outcomes (bottom left) and clinical efficacy (bottom right) at 3 years according to frailty. Stacked bar graphs show the causes of failed efficacy using a hierarchical approach (death, stroke, hospitalization, and KCCQ <45 or KCCQ decrease >10 points from baseline, in that order). ∗Hospitalization for valve-Related dysfunction or heart failure. KCCQ = Kansas City Cardiomyopathy Questionnaire; STS-PROM = Society of Thoracic Surgeons Predicted Risk of Mortality; TAVR = transcatheter aortic valve replacement.

### Patient-reported health status

**Figure 1:**
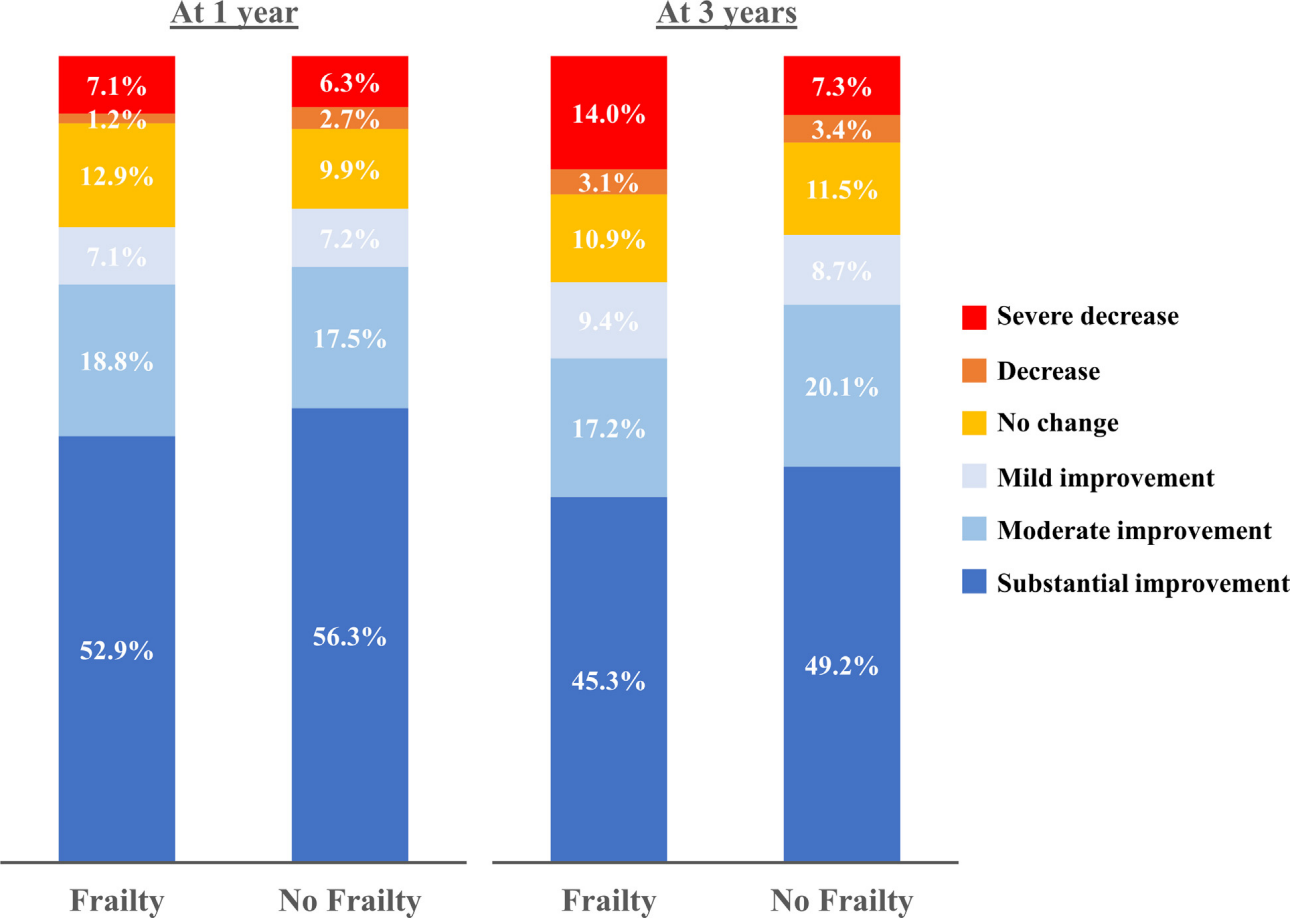
**Ordinal Analysis of Overall Kansas CityCardiomyopathy Questionnaire Score According to Frailty**

Changes in the overall KCCQ score and its constituent domains throughout the study period are summarized in Table 2. Completion rates of KCCQ assessment for surviving patients with and without frailty at baseline were 94.2% and 94.3% prior to the procedure, 82.9% and 87.8% at 30 days, 83.8% and 87.9% at 1 year, and 73.8% and 78.0% at 3 years, respectively. At baseline, frail patients had lower KCCQ scores in all four domains resulting in a lower overall KCCQ score than those without frailty. This difference remained consistent throughout the study period, including after TAVR (overall median [Q1, Q3] KCCQ score at baseline: 49.0 [31.9, 63.5] vs 56.2 [41.0, 75.0], differences 8.3% [3.7%-12.5%], *P* < 0.001; 30 days: 69.8 [54.7, 86.3] vs 83.3 [69.8, 91.7], differences 9.9% [5.4%-14.6%], *P* < 0.001; 1 year: 76.0 [61.7, 90.4] vs 86.5 [70.8, 93.8], differences 6.2% [2.1%-10.4%], *P* = 0.001; 3 years: 70.8 [Q1, Q3: 53.1, 85.4] vs 83.3 [68.2, 92.2], differences 10.4% [5.2%-15.6%], *P* < 0.001) (Central Illustration). Improvements (change from baseline) in overall KCCQ score were similar after TAVR in patients with and without frailty (1 year: 25.8 ± 27.0 vs 23.5 ± 22.5, difference 2.3 [−3.1 to 7.7], *P* = 0.40; 3 years: 18.8 ± 26.3 vs 20.8 ± 21.6, difference −2.05 [−8.0 to 3.9], *P* = 0.50, respectively) accompanied by a similar distribution in ordinal analysis, with more than 70% and 60% of patients, respectively, having at least a mild improvement in overall KCCQ score at 1 and 3 year follow-up (Figure 1 and Table 3).

**Table 2:** KCCQ Score Over Time

	All Patients (N = 739)	Patients With Frailty (n = 122)	Patients Without Frailty (n = 617)	Hodges-Lehmann Median Difference (95% CI)	*P* Value
At baseline	N = 697	n = 115	n = 582		
KCCQ overall score	54.7 (39.6-71.9)	49.0 (31.9-63.5)	56.2 (41.0-72.5)	8.3% (3.7%-12.5%)	<0.001
Physical limitation	58.3 (33.3-75.0)	41.7 (25.0-66.7)	58.3 (41.6-75.0)	8.3% (8.3%-16.7%)	<0.001
Quality of life	50.0 (25.0-62.5)	50.0 (25.0-62.5)	50.0 (25.0-62.5)	0.0% (0.0%-12.5%)	0.29
Social limitation	58.3 (33.3-83.3)	50.0 (25.0-75.0)	58.3 (33.3-83.3)	8.3% (0.0%-16.6%)	0.046
Symptom frequency	64.6 (46.9-79.2)	56.2 (33.3-75.0)	66.7 (50.0-79.2)	10.4% (4.2%-14.6%)	<0.001
At 30 d	N = 633	n = 97	n = 536		
KCCQ overall score	81.8 (67.7-91.7)	69.8 (54.7-86.3)	83.3 (69.8-91.7)	9.9% (5.4%-14.6%)	<0.001
Physical limitation	83.3 (58.3-100.0)	66.7 (41.6-87.5)	83.3 (58.3-100.0)	12.5% (8.3%-16.7%)	<0.001
Quality of life	87.5 (62.5-100.0)	75.0 (50.0-87.5)	87.5 (75.0-100.0)	12.5% (0.0%-12.5%)	0.001
Social limitation	83.3 (66.7-100.0)	75.0 (58.3-91.7)	87.5 (66.7-100.0)	8.3% (0.0%-16.7%)	<0.001
Symptom frequency	83.3 (68.8-95.8)	75.0 (61.5-85.4)	83.3 (70.8-95.8)	8.3% (4.2%-12.5%)	<0.001
At 1 y	N = 584	n = 88	n = 496		
KCCQ overall score	85.4 (68.9-93.7)	76.0 (61.7-90.4)	86.5 (70.8-93.8)	6.2% (2.1%-10.4%)	0.001
Physical limitation	83.3 (58.3-100.0)	75.0 (50.0-91.7)	83.3 (58.3-100.0)	8.3% (0.0%-16.7%)	0.031
Quality of life	87.5 (75.0-100.0)	75.0 (62.5-100.0)	87.5 (75.0-100.0)	0.0% (0.0%-12.5%)	0.006
Social limitation	91.7 (66.7-100.0)	75.0 (58.3-91.7)	91.7 (66.7-100.0)	8.3% (0.0%-16.7%)	<0.001
Symptom frequency	83.3 (70.8-100.0)	81.2 (68.8-95.8)	83.3 (72.9-100.0)	4.2% (0.0%-8.3%)	0.086
At 3 y	N = 441	n = 65	n = 376		
KCCQ overall score	81.2 (66.7-91.7)	70.8 (53.1-85.4)	83.3 (68.2-92.2)	10.4% (5.2%-15.6%)	<0.001
Physical limitation	83.3 (58.3-91.7)	75.0 (40.6-87.5)	83.3 (58.3-91.7)	8.3% (0.0%-16.7%)	0.003
Quality of life	75.0 (62.5-100.0)	62.5 (50.0-87.5)	87.5 (75.0-100.0)	12.5% (12.5%-12.5%)	<0.001
Social limitation	83.3 (66.7-100.0)	75.0 (52.1-91.7)	83.3 (66.7-100.0)	8.3% (0.0%-16.7%)	<0.001
Symptom frequency	83.3 (70.8-95.8)	80.2 (58.3-94.8)	83.3 (72.9-95.8)	4.2% (0.0%-8.3%)	0.034

Values are median (25%-75% interquartile) unless otherwise indicated.

KCCQ = Kansas City Cardiomyopathy Questionnaire.

**Table 3:** Changes in Health Status Compared to Baseline Overall KCCQ Score

	All Patients (N = 739)	Patients With Frailty (N = 122)	Patients Without Frailty (N = 617)	Difference (95% CI)	*P* Value
At 1 y	N = 584	n = 88	n = 496		
Change in KCCQ overall score	23.8 ± 23.2	25.8 ± 27.0	23.5 ± 22.5	2.32 (−3.05 to 7.70)	0.40
Severe decrease	36 (6.4%)	6 (7.1%)	30 (6.3%)	1% (−5% to 6%)	0.81
Decrease	14 (2.5%)	1 (1.1%)	13 (2.7%)	−2% (−5% to 2%)	0.71
No change	58 (10.4%)	11 (12.5%)	47 (9.9%)	3% (−4% to 10%)	0.44
Mildly improved	40 (7.2%)	6 (6.8%)	34 (7.2%)	−0% (−6% to 6%)	1.00
Moderately improved	99 (17.7%)	16 (18.2%)	83 (17.5%)	1% (−8% to 10%)	0.76
Substantially improved	312 (55.8%)	45 (51.1%)	267 (56.3%)	−3% (−15% to 8%)	0.64
At 3 y	N = 441	n = 65	n = 376		
Change in KCCQ overall score	20.5 ± 22.3	18.8 ± 26.3	20.8 ± 21.6	−2.05 (−8.00 to 3.91)	0.50
Severe decrease	35 (8.3%)	9 (14.0%)	26 (7.3%)	7% (−0% to 14%)	0.083
Decrease	14 (3.3%)	2 (3.1%)	12 (3.4%)	−0% (−5% to 5%)	1.00
No change	48 (11.4%)	7 (10.9%)	41 (11.5%)	−0% (−9% to 8%)	1.00
Mildly improved	37 (8.8%)	6 (9.4%)	31 (8.7%)	1% (−7% to 8%)	0.81
Moderately improved	83 (19.7%)	11 (17.2%)	72 (20.1%)	−3% (−14% to 8%)	0.73
Substantially improved	205 (48.6%)	29 (45.3%)	176 (49.2%)	−4% (−17% to 9%)	0.59

Values are mean ± SD or n (%) unless otherwise indicated.

Ordinal analysis of KCCQ was performed according to change in overall KCCQ score from baseline: severe decrease (decrease by >10 points); decrease (decrease by 5-10 points); no change (change between −5 and 5 points); mildly improved (increase by 5-10 points); moderately improved (increase by 10-20 points); substantially improved (increase by ≥20 points).

Abbreviations as in Table 2.

### Clinical outcomes

**Figure 2:**
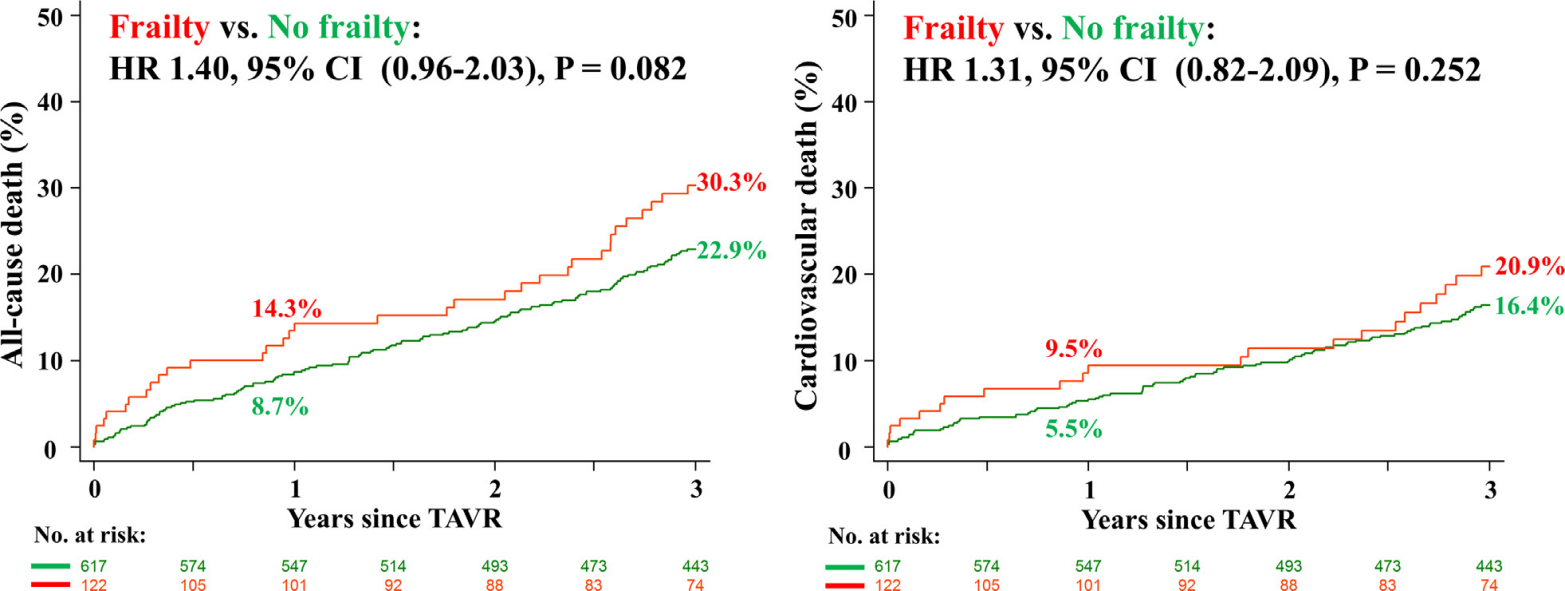
**Cumulative Incidence Curves for All-Cause and Cardiovascular Mortality According to Frailty** Abbreviations as in Central Illustration.

Table 4 shows clinical outcomes according to frailty. The rates of all-cause and cardiovascular death were 4.2% and 3.3%, 14.3% and 9.5%, and 30.3% and 20.9% in frail patients and 1.1% and 1.1%, 8.7% and 5.5%, and 22.9% and 16.4% of nonfrail controls at 30 days, 1, and 3 years, respectively. All-cause death was higher in frail patients after TAVR but the difference was not statistically significant (1 year: HR: 1.71, 95% CI: 0.99-2.95, *P* = 0.055; 3 years: HR: 1.40, 95% CI: 0.96-2.03, *P* = 0.082) (Figure 2). There was no difference in the frequency of stroke or hospitalization for valve-related dysfunction or heart failure compared with nonfrail controls. These results were consistent in the competing risk survival analysis (Supplemental Table 1).

**Table 4:** Outcomes According to Frailty

	Patients With Frailty (N = 122)	Patients Without Frailty (N = 617)	Hazard or Risk Ratio (95% CI)^a^	*P* Value
30-d outcomes^b^				
Overall mortality	5/122 (4.2%)	7/617 (1.1%)	3.68 (1.17-11.6)	0.026
Cardiovascular mortality	4/122 (3.3%)	7/617 (1.1%)	2.94 (0.86-10.1)	0.085
All stroke	2/122 (1.7%)	16/617 (2.6%)	0.63 (0.15-2.75)	0.54
Hospitalization^c^	1/122 (0.9%)	8/617 (1.3%)	0.65 (0.08-5.17)	0.68
1-y outcomes^b^				
Clinical efficacy	71/103 (68.9%)	390/530 (73.6%)	0.94 (0.82-1.08)	0.33
Favorable outcome	67/103 (65.0%)	398/530 (75.1%)	0.87 (0.75-1.01)	0.035
Acceptable outcome	11/103 (10.7%)	31/530 (5.8%)	1.83 (0.95-3.52)	0.071
Unfavorable outcome	25/103 (24.3%)	101/530 (19.1%)	1.27 (0.87-1.86)	0.23
Overall mortality	17/122 (14.3%)	53/617 (8.7%)	1.71 (0.99-2.95)	0.055
Cardiovascular mortality	11/122 (9.5%)	33/617 (5.5%)	1.77 (0.89-3.50)	0.10
All stroke	4/122 (3.5%)	28/616 (4.7%)	0.74 (0.26-2.11)	0.57
Hospitalization^c^	15/122 (13.6%)	54/616 (9.2%)	1.51 (0.85-2.67)	0.16
3-y outcomes^b^				
Clinical efficacy	45/98 (45.9%)	277/493 (56.2%)	0.82 (0.65-1.03)	0.062
Favorable outcome	43/98 (43.9%)	298/493 (60.4%)	0.73 (0.58-0.92)	0.002
Acceptable outcome	8/98 (8.2%)	24/493 (4.9%)	1.68 (0.78-3.63)	0.19
Unfavorable outcome	47/98 (48.0%)	171/493 (34.7%)	1.38 (1.09-1.75)	0.013
Overall mortality	34/122 (30.3%)	135/617 (22.9%)	1.40 (0.96-2.03)	0.082
Cardiovascular mortality	22/122 (20.9%)	93/617 (16.4%)	1.31 (0.82-2.09)	0.25
All stroke	6/122 (5.2%)	35/616 (5.8%)	0.87 (0.37-2.08)	0.76
Hospitalization^c^	22/122 (21.3%)	88/616 (16.0%)	1.42 (0.89-2.27)	0.14

Values are counts of events (first occurrence per patient only, % from Kaplan-Meier estimates, Cox model with HR, and Wald test for statistical significance of the HR) for mortality, stroke, and hospitalization.

Clinical efficacy: Freedom from all-cause mortality AND Freedom from all stroke AND Freedom from hospitalization for procedure- or valve-related causes AND Freedom from Unfavorable outcome; Favorable outcome: a patient is alive, AND has a KCCQ Overall Summary score ≥60, AND has not had a decline of >10 points in the KCCQ Overall Summary score from baseline; Acceptable outcome: [a patient is alive, AND has an overall KCCQ score ≥45, AND has not had a decline of >10 points in the overall KCCQ score from baseline; Unfavorable outcome: [a patient is not alive] OR [is alive AND has either an overall KCCQ Overall score <45 OR has not had a decline of >10 points in the overall KCCQ score from baseline.

Abbreviations as in Table 2.

a Frailty vs no frailty.

b N = 8 patients withdrew consent before 30 days and no clinical outcomes were recorded (n = 2 frail and n = 6 not frail patients). n = 106 alive patients did not have KCCQ score at baseline or 1-year follow-up so are not in the denominator; n = 148 alive patients did not have KCCQ score at baseline or 3-year follow-up so are not in the denominator.

c Hospitalization for valve-related dysfunction or heart failure.

### Integrated clinical outcome and patient-reported health status endpoints

Integrated clinical outcomes and patient-reported health status endpoints were assessed in 84.4% and 77.9% of frail patients and 85.9% and 79.9% of nonfrail patients at 1 and 3 years, respectively (Table 4). An overall favorable outcome was achieved in 65.0% of frail patients vs 75.1% of nonfrail patients at 1 year, and in 43.9% of frail patients vs 60.4% of nonfrail patients at 3 years. Furthermore, at 3 years after TAVR, frailty was associated with an increased risk of unfavorable outcomes (RR: 1.38 [95% CI: 1.09-1.75]; *P* = 0.013). At the same time, we observed a reduced clinical efficacy in frail patients though not statistically significant (RR: 0.82 [95% CI: 0.65-1.03]; *P* = 0.062) (Central Illustration). In the sensitivity analysis using multiple imputation for missing variables, patients with frailty had an increased risk of VARC-3 unfavorable outcomes at 3 years following TAVR, consistent with the findings of the main analysis, but the difference was not statistically significant (Supplemental Table 2).

### Full factorial model

Baseline characteristics and outcomes according to the presence of frailty and type of THV were consistent in the intention-to-treat and per-protocol populations. There was no significant interaction between frailty and the type of THV for all reported endpoints (Supplemental Tables 3 to 5).

### Exploratory analysis

We performed an exploratory analysis stratifying patients according to baseline frailty and favorable, acceptable, and unfavorable outcomes to examine the association between individual characteristics and general outcomes in those undergoing VARC 3 assessment at 3 years (98 frail and 493 nonfrail patients, respectively). In this analysis, male sex and lower STS-PROM were associated with VARC-3 favorable outcome, whereas the presence of comorbidities, low activity, and malnutrition were associated with unfavorable outcome in nonfrail patients. In contrast, only the presence of comorbidities was associated with unfavorable outcome in frail patients (Supplemental Table 6).

## Discussion

The main findings of the present study can be summarized as follows: 1) more than 1 in 6 patients with severe AS undergoing TAVR were deemed to be frail; 2) these patients were more likely to be female and experience symptoms of advanced heart failure before TAVR; 3) whilst frail patients had consistently lower overall KCCQ scores throughout the study period, improvements after TAVR were comparable in frail and nonfrail patients; and 4) risks of all-cause mortality and unfavorable outcome after TAVR are increased in frail patients and associated with reduced clinical efficacy at 3-year follow-up.

A large proportion of patients with valvular heart disease are older and present with disease-specific symptoms accompanied by signs of physical and nonphysical frailty. Clinically relevant AS is associated with comorbidities that may facilitate the development of frailty through various mechanisms, such as physical limitation and disease-specific symptoms.^1^^,^^17^^,^^32^ The reported prevalence of frailty in patients undergoing AVR varies widely between studies due to differences in patient populations and varying definitions of frailty. The prospective FRAILTY-AVR (Frailty Assessment Before Cardiac Surgery & Transcatheter Interventions) study assessed frailty in patients with severe AS undergoing TAVR or SAVR using seven different frailty scales and found that the prevalence ranged from 35% to 74% in the TAVR group and from 12% to 56% in the SAVR group, depending on the definition used. The Essential Frailty Toolset, integrating lower-extremity weakness, cognitive impairment, anemia, and hypoalbuminemia, outperformed the other scales and was recommended for use in this setting.^14^ In the SCOPE I trial, frailty was evaluated by a comprehensive geriatric assessment including physical, cognitive, nutritional, and endurance dimensions. Although our frailty assessment approach has yet to be validated in other cohorts, it aligns with the comprehensive geriatric assessment strategy shown to be promising in the FRAILTY-AVR study. The prevalence of frailty is likely to be lower in younger and healthier populations. More recent low to intermediate risk cohorts of the PARTNER (Placement of Aortic Transcatheter Valves) trials and S3i registry assessed frailty using a 4-domain scale and reported that two-thirds of patients were prefrail, whilst only 6.1% met criteria for physical frailty.^33^ In the SCOPE I trial, the prevalence of frail patients was only 16.5%, perhaps reflecting healthy volunteer bias frequently encountered in randomized studies.

Of note in the present analysis, female sex was more frequently observed in the frail group, which may reflect different disease progression and later diagnosis of AS in women,^9^^,^^33^, ^34^, ^35^, ^36^, ^37^ whereas the age distribution, common comorbidities, surgical risk, and echocardiographic features were similar between frail and nonfrail patients. The overlap of frailty with other comorbidities introduces complexity in distinguishing the independent effects of frailty. Indeed, frailty may serve both as a separate predictor and as an amplifying factor for other high-risk conditions, as seen in our exploratory analyses. This may highlight a critical challenge in frailty research: comorbidities such as cardiovascular and metabolic disorders often coexist with frailty, making it difficult to isolate the specific contribution of frailty to outcomes. These findings suggest that current methods of patient risk stratification that are mainly based on surgical risk scores and anatomical features may underappreciate the impact of frailty in patients undergoing TAVR.^9^^,^^38^ Integrated assessment of surgical risk as well as frailty may be key in identifying patient-specific risks and tailoring optimal treatment for the individual patient.^1^^,^^3^

It is well known that frail patients have worse health status and overall prognosis compared to nonfrail patients, even after AVR.^3^^,^^9^^,^^10^^,^^33^^,^^38^ Thus, in the pooled analysis of the PARTNER trials and S3i registry, frailty was associated with an increased risk of 2-year death and a lower KCCQ score. Of note, the negative impact on clinical and patient-reported outcomes was observed even in the prefrail state.^33^ Consistent with previous studies, we observed a signal of higher all-cause mortality and lower KCCQ scores in frail patients up to 3 years of follow-up. In contrast, the extent of improvement of the overall KCCQ score was similar in frail and nonfrail patients, and a significant proportion of frail patients achieved at least mild improvement. Importantly, this improvement may justify invasive treatment strategies in patients with reduced health status and frailty at baseline even in the absence of an effect on mortality.^20^^,^^39^ Patients with a poor quality of life due to severe AS gain longevity and improved health status after TAVR. However, it should be noted that assessment of health status can potentially be biased over follow-up since patients who derive less benefit may also be more likely to die. Moreover, improvement in health status alone may not be an accurate reflection of truly good health. Whilst survival without deterioration in quality of life is the treatment goal for patients with good baseline health status, survival and the achievement of a reasonable quality of life may be sufficient for patients with poor overall health status.^20^ The ultimate goal of invasive treatment must be the extension of life and good health status, and composite endpoints that integrate survival, change in health status, and actual health status provide a more interpretable assessment of overall benefit.

The VARC definitions provide a reproducible and standardized tool to compare clinical endpoints in TAVR and SAVR trials and the recently updated VARC-3 criteria will be instrumental to the design of future trials. Endpoints that integrate clinical outcomes and patient-reported health status (VARC-3 favorable, acceptable, and unfavorable outcomes and clinical efficacy) fully capture treatment utility at the individual patient level.^19^^,^^20^^,^^26^ In the present analysis, frail patients had a higher rate of unfavorable outcomes and a trend toward reduced clinical efficacy over 3 years of follow-up after TAVR. Poor outcomes (defined as death, overall KCCQ score <60, or reduction in overall KCCQ score >10 from baseline) were also more frequent 1 year after TAVR in frail patients (50.0% vs 31.5%, log-rank *P* = 0.004) in a similar previous study.^9^ These results suggest that TAVR may be futile in up to 50% of all frail patients due to reduced survival and/or poor postprocedural health status. Further research is warranted to determine the utility of less invasive treatment in this population.

Although frail patients have generally been considered to benefit from a less invasive approach by means of TAVR rather than SAVR,^40^^,^^41^ recent analyses from the PARTNER trials and pooled outcomes of the CoreValve High Risk and SURTAVI (Safety and Efficacy Study of the Medtronic CoreValve® System in the Treatment of Severe, Symptomatic Aortic Stenosis in Intermediate Risk Subjects Who Need Aortic Valve Replacement) trials suggest that frailty is not associated with a differential impact of treatment modality on mortality and disease-specific health status.^13^^,^^33^ Our analysis suggests that frail patients may derive less benefit from TAVR in terms of VARC-3 favorable outcome and clinical efficacy. Importantly, the negative impact of frailty may not be mitigated by advances in device technology, accumulating operator experience and high procedural success. In the present analysis, patients with and without frailty has a similar rate of procedural success and periprocedural complications. These findings may have important implications for the timing of intervention. Current guidelines recommend that AVR should be considered at the onset of symptoms in patients with severe AS.^40^^,^^41^ However, given the interdependence between progression of AS and the development of frailty, patients with at least one marker of frailty might benefit from earlier intervention before the onset of symptoms or when AS is less severe. Recently, several clinical trials have evaluated the effects of early intervention in patients with asymptomatic severe and moderate AS, while additional trials are underway to extend these findings (ESTIMATE [Early Surgery for Patients With Asymptomatic Aortic Stenosis] [NCT02627391], EVOLVED Surgical AVR [MRI in Randomised Cohorts of Asymptomatic AS] [NCT05178368], EASY-AS [The Early Valve Replacement in Severe ASYmptomatic Aortic Stenosis Study] [NCT04204915], PROGRESS [Management of Moderate Aortic Stenosis by Clinical Surveillance or TAVR] [NCT04889872], and EXPAND TAVI [Evolut EXPAND TAVR II Pivotal Trial [NCT05149755]).^42^, ^43^, ^44^, ^45^, ^46^ There results will have significant implications the future management of patients with AS.

### Study limitations

The results of the present study should be interpreted in the light of several limitations. First, although we provide comprehensive data from an established randomized control trial with high data quality standards and independent event adjudication, a modest proportion of patients did not have KCCQ scores assessed during the study period. This underreporting of KCCQ scores may have introduced responder bias, as sicker patients are less likely to complete questionnaires.^26^ Indeed, in the present analysis, the completion of KCCQ assessment was relatively lower in the frail group compared with that in the nonfrail group. Purposeful approaches to minimize missing data from patient-reported outcome instruments should be considered.^47^ Second, although frailty status was determined during the screening process based upon prespecified definitions, assessment was performed by the heart team at each participating center. Furthermore, the database collected overall frailty scores rather than individual parameters. The results may therefore be confounded by unmeasured variables and must be interpreted with caution. Third, we could not assess frailty according to other definitions which may influence its prevalence and impact. Similarly, we were unable to assess frailty status immediately after TAVR which may improve with initial relief of mechanical obstruction or progress during subsequent follow-up, thereby diluting the observation. Fourth, this subgroup analysis of the SCOPE I cohort was not prespecified and not powered to detect differences in clinical outcomes between patients with and without baseline frailty. Therefore, the present findings cannot conclusively determine the impact of frailty on patient outcomes in the TAVR population and are hypothesis generating. Finally, the present cohort was predominantly composed of octogenarians, and our results may not be applicable to younger patients with less comorbidities and longer life expectancy.

## Conclusions

In the SCOPE I randomized controlled trial, more than one in six patients with severe AS undergoing TAVR were deemed to be frail. These patients demonstrated similar improvement in overall KCCQ score after TAVR. However, frailty was associated with an increased risk of VARC-3 unfavorable outcome and had a trend toward increased all-cause mortality and reduced clinical efficacy throughout 3 years of follow-up.

Perspectives **COMPETENCY IN MEDICAL KNOWLEDGE:** Frailty is common in patients with severe AS undergoing TAVR and associated with increased risk of mortality and poor health status. Although TAVR is less invasive than SAVR and considered to be a better treatment option for this population, assessment of its utility in this population is complex. In the present post-hoc analysis of the SCOPE I randomized trial, frail patients demonstrated a similar improvement in overall KCCQ score after TAVR, but a higher frequency of unfavorable outcomes throughout 3 years of follow-up, suggesting that they may benefit less from TAVR than their non-frail counterparts.

**TRANSLATIONAL OUTLOOK:** Further studies are warranted to determine the benefits of earlier intervention in patients with AS before frailty is established.

## Funding support and author disclosures

SCOPE I was funded by Boston Scientific, Marlborough, MA. Dr Lanz reports speaker fees to the institution from Edwards Lifesciences and Abbott and served as advisory board member for Abbott. Dr Prendergast reports speaker fees from Edwards Lifesciences and serves as a steering/an executive committee group member for Medtronic and Valvosoft. Dr Windecker reports research, travel, or educational grants to the institution without personal remuneration from Abbott, Abiomed, Amgen, Astra Zeneca, Bayer, Braun, Biotronik, Boehringer Ingelheim, Boston Scientific, Bristol Myers Squibb, Cardinal Health, CardioValve, Cordis Medical, Corflow Therapeutics, CSL Behring, Daiichi Sankyo, Edwards Lifesciences, Farapulse Inc Fumedica, Guerbet, Idorsia, Inari Medical, InfraRedx, Janssen-Cilag, Johnson & Johnson, Medalliance, Medicure, Medtronic, Merck Sharp & Dohm, Miracor Medical, MonarQ, Novartis, Novo Nordisk, Organon, OrPha Suisse, Pharming Tech. Pfizer, Polares, Regeneron, Sanofi-Aventis, Servier, Sinomed, Terumo, Vifor, V-Wave; .has served as advisory board member and/or member of the steering/executive group of trials funded by Abbott, Abiomed, Amgen, Astra Zeneca, Bayer, Boston Scientific, Biotronik, Bristol Myers Squibb, Edwards Lifesciences, MedAlliance, Medtronic, Novartis, Polares, Recardio, Sinomed, Terumo, and V-Wave with payments to the institution but no personal payments; is also member of the steering/executive committee group of several investigator-initiated trials that receive funding by industry without impact on his personal remuneration. Dr Pilgrim reports research, travel, or educational grants to the institution without personal remuneration from Biotronik, Boston Scientific, Edwards Lifesciences, Medtronic and ATSens; speaker fees and consultancy fees to the institution from Biotronik, Boston Scientific, Edwards Lifesciences, Abbott, Medtronic, Biosensors, and Highlife. All other authors have reported that they have no relationships relevant to the contents of this paper to disclose.
