# Supplementary material for: The Impact of Frailty on VARC-3 Integrated Outcomes in Patients Undergoing Transcatheter Aortic Valve Replacement
Source: JACC Adv. 2025 Feb 14;4(3):101594. doi: 10.1016/j.jacadv.2025.101594 (PMC11872499; doi:10.1016/j.jacadv.2025.101594)
Supplement: Supplemental Tables 1-6 [file mmc1.docx]

## **Supplemental Table 1.** Clinical outcomes according to frailty in a competing risk analysis

|  | **Frailty vs. No frailty** | **P value** |
| --- | --- | --- |
|  | **subdistribution hazard ratio (95% CI)** |  |
| **1-year clinical outcomes** |  |  |
| Cardiovascular mortality | 1.75 (0.89-3.47) | 0.11 |
| All stroke | 0.73 (0.26-2.06) | 0.55 |
| Hospitalization for valve-related dysfunction or heart failure | 1.46 (0.82-2.58) | 0.20 |
| **3-year outcomes** |  |  |
| Cardiovascular mortality | 1.30 (0.82-2.07) | 0.27 |
| All stroke | 0.87 (0.37-2.07) | 0.76 |
| Hospitalization for valve-related dysfunction or heart failure | 1.37 (0.86-2.19) | 0.19 |

## **Supplemental Table 2.** VARC-3 integrated outcomes according to frailty after multiple imputations of missing data

|  | **Frailty** | **No frailty** | **Frailty vs. No frailty** | **P value** |
| --- | --- | --- | --- | --- |
|  | **N = 122**  **Proportion (95% CI)** | **N = 617**  **Proportion (95% CI)** | **Risk ratio (95% CI)** |  |
| **1-year outcomes** |  |  |  |  |
| Clinical efficacy | 69.8% (61.2%-78.4%) | 73.6% (69.9%-77.2%) | 0.95 (0.75-1.20) | 0.66 |
| Favorable outcome | 67.3% (58.5%-76.1%) | 75.3% (71.8%-78.9%) | 0.89 (0.70-1.13) | 0.35 |
| Acceptable outcome | 9.8% (4.2%-15.5%) | 5.6% (3.7%- 7.5%) | 1.76 (0.88-3.53) | 0.11 |
| Unfavorable outcome | 23.0% (15.2%-30.8%) | 19.1% (15.9%-22.4%) | 1.20 (0.79-1.83) | 0.40 |
| **3-year outcomes** |  |  |  |  |
| Clinical efficacy | 45.4% (35.4%-55.4%) | 54.2% (49.9%-58.4%) | 0.84 (0.62-1.14) | 0.25 |
| Favorable outcome | 45.2% (35.4%-55.1%) | 59.4% (55.2%-63.7%) | 0.76 (0.56-1.03) | 0.073 |
| Acceptable outcome | 7.4% (2.4%-12.4%) | 4.5% (2.8%-6.3%) | 1.62 (0.73-3.59) | 0.23 |
| Unfavorable outcome | 47.3% (37.2%-57.4%) | 36.1% (31.9%-40.3%) | 1.31 (0.96-1.78) | 0.086 |
| Multiple imputation of missing data using chained equations, estimates combined from 20 data-sets using Rubin's rule. The imputed variables included: aortic valve area, moderate or severe aortic/mitral/tricuspid regurgitation, baseline serum albumin, mode of transfemoral access, device type and size, moderate or severe prosthetic valve regurgitation, and KCCQ scores and clinical outcomes at 30 days, 1 year, and 3 years. Non-missing baseline variables incorporated into the imputation model included age, sex, body mass index, Society of Thoracic Surgeons Predicted Risk of Mortality score, left ventricular ejection fraction, New York Heart Association class III/IV, Canadian Cardiovascular Society angina grade 3/4, and comorbidities (hypertension, diabetes, creatinine >2 mg/dL, coronary artery disease, chronic obstructive pulmonary disease, atrial fibrillation, extracardiac arteriopathy, dementia/cognitive impairment, and history of myocardial infarction, stroke, or syncope). | | | | |

## **Supplemental Table 3.** Baseline characteristics according to frailty and device type

|  | **Intention-to-treat population** | | | | | | **Per-protocol population** | | | | | |
| --- | --- | --- | --- | --- | --- | --- | --- | --- | --- | --- | --- | --- |
|  | **SAPIEN 3** | | | **Acurate Neo** | | | **SAPIEN 3** | | | **Acurate Neo** | | |
|  | **Frailty**  **N = 54** | **No frailty**  **N = 313** | **P value** | **Frailty**  **N = 68** | **No frailty**  **N = 304** | **P value** | **Frailty**  **N = 53** | **No frailty**  **N = 315** | **P value** | **Frailty**  **N = 67** | **No frailty**  **N = 297** | **P value** |
| Age, years | 82.6 ± 3.7 | 83.1 ± 4.0 | 0.32 | 82.1 ± 4.0 | 82.7 ± 4.3 | 0.27 | 82.8 ± 3.7 | 83.0 ± 4.0 | 0.70 | 82.0 ± 3.9 | 82.8 ± 4.3 | 0.15 |
| Female, n (%) | 38 (70%) | 164 (52%) | 0.017 | 44 (65%) | 174 (57%) | 0.28 | 37 (70%) | 165 (52%) | 0.025 | 44 (66%) | 170 (57%) | 0.22 |
| Body mass index, kg/cm² | 28.1 ± 4.9 | 27.8 ± 4.6 | 0.74 | 27.6 ± 4.4 | 27.3 ± 4.4 | 0.62 | 28.1 ± 5.0 | 27.9 ± 4.6 | 0.79 | 27.5 ± 4.5 | 27.2 ± 4.4 | 0.58 |
| STS-PROM, % | 4.5 ± 2.6 | 4.2 ± 2.7 | 0.46 | 4.8 ± 5.1 | 4.2 ± 2.4 | 0.12 | 4.5 ± 2.7 | 4.2 ± 2.7 | 0.60 | 4.9 ± 5.1 | 4.1 ± 2.4 | 0.085 |
| NYHA III or IV, n (%) | 43 (80%) | 225 (72%) | 0.32 | 61 (90%) | 225 (74%) | 0.004 | 42 (79%) | 226 (72%) | 0.32 | 61 (91%) | 220 (74%) | 0.002 |
| CCS grade III or IV, n (%) | 3 (6%) | 20 (6%) | 1.0 | 3 (4%) | 18 (6%) | 0.78 | 2 (4%) | 20 (6%) | 0.75 | 4 (6%) | 18 (6%) | 1.0 |
| Syncope, (%) | 8 (15%) | 36 (12%) | 0.50 | 7 (10%) | 30 (10%) | 1.0 | 8 (15%) | 36 (11%) | 0.49 | 7 (10%) | 30 (10%) | 1.0 |
| **Comorbidities** |  |  |  |  |  |  |  |  |  |  |  |  |
| Hypertension, n (%) | 51 (94%) | 282 (90%) | 0.45 | 61 (90%) | 280 (92%) | 0.48 | 50 (94%) | 284 (90%) | 0.45 | 60 (90%) | 273 (92%) | 0.48 |
| Diabetes mellitus, n (%) | 17 (31%) | 99 (32%) | 1.0 | 18 (26%) | 90 (30%) | 0.66 | 16 (30%) | 100 (32%) | 0.87 | 18 (27%) | 85 (29%) | 0.88 |
| Creatinine concentration >2 mg/dL, n (%) | 1 (2%) | 12 (4%) | 0.70 | 3 (4%) | 12 (4%) | 0.74 | 1 (2%) | 13 (4%) | 0.70 | 3 (4%) | 11 (4%) | 0.73 |
| Coronary artery disease, n (%) | 31 (57%) | 188 (60%) | 0.77 | 38 (56%) | 180 (59%) | 0.68 | 30 (57%) | 190 (60%) | 0.65 | 37 (55%) | 175 (59%) | 0.59 |
| Previous myocardial infarction, n (%) | 9 (17%) | 38 (12%) | 0.38 | 9 (13%) | 30 (10%) | 0.39 | 9 (17%) | 40 (13%) | 0.39 | 8 (12%) | 27 (9%) | 0.49 |
| Previous stroke or TIA, n (%) | 9 (17%) | 38 (12%) | 0.38 | 13 (19%) | 34 (11%) | 0.10 | 8 (15%) | 37 (12%) | 0.50 | 14 (21%) | 33 (11%) | 0.042 |
| COPD, n (%) | 9 (17%) | 35 (11%) | 0.26 | 6 (9%) | 27 (9%) | 1.0 | 9 (17%) | 36 (11%) | 0.26 | 6 (9%) | 26 (9%) | 1.0 |
| Atrial fibrillation, n (%) | 23 (43%) | 114 (36%) | 0.45 | 27 (40%) | 107 (35%) | 0.49 | 21 (40%) | 115 (37%) | 0.76 | 28 (42%) | 104 (35%) | 0.33 |
| Extracardiac arteriopathy, n (%) | 7 (13%) | 33 (11%) | 0.64 | 6 (9%) | 40 (13%) | 0.42 | 6 (11%) | 33 (10%) | 0.81 | 6 (9%) | 39 (13%) | 0.42 |
| **Echocardiography*** |  |  |  |  |  |  |  |  |  |  |  |  |
| Aortic valve area, cm² | 0.73 ± 0.16 | 0.73 ± 0.19 | 0.82 | 0.68 ± 0.21 | 0.73 ± 0.20 | 0.12 | 0.73 ± 0.17 | 0.73 ± 0.19 | 0.76 | 0.69 ± 0.21 | 0.73 ± 0.20 | 0.16 |
| Mean aortic valve gradient, mmHg | 40.20 ± 13.19 | 41.70 ± 15.42 | 0.50 | 45.0 ± 20.4 | 42.4 ± 16.4 | 0.25 | 40.5 ± 13.7 | 41.7 ± 15.4 | 0.59 | 44.9 ± 20.3 | 42.5 ± 16.5 | 0.30 |
| Left ventricular ejection fraction, % | 56.8 ± 9.5 | 57.2 ± 11.0 | 0.83 | 54.3 ± 11.8 | 56.8 ± 10.9 | 0.091 | 57.5 ± 9.6 | 57.2 ± 10.8 | 0.83 | 54.3 ± 11.4 | 57.0 ± 10.9 | 0.073 |
| Moderate or severe aortic regurgitation, n (%) | 6 (13%) | 35 (12%) | 0.81 | 9 (15%) | 28 (10%) | 0.26 | 7 (15%) | 34 (12%) | 0.47 | 8 (14%) | 28 (10%) | 0.49 |
| Moderate or severe mitral regurgitation, n (%) | 9 (17%) | 50 (16%) | 0.84 | 9 (13%) | 43 (14%) | 1.0 | 9 (17%) | 53 (17%) | 1.0 | 9 (13%) | 39 (13%) | 1.0 |
| Moderate or severe tricuspid regurgitation, n (%) | 6 (11%) | 38 (13%) | 1.0 | 7 (11%) | 31 (11%) | 1.0 | 6 (11%) | 40 (13%) | 0.83 | 7 (11%) | 29 (10%) | 0.82 |
| Depicted are counts (%) or means ± standard deviations with sample sizes.  *Transthoracic echocardiography used, if not available transesophageal echocardiography used; if no echocardiography available, catheterization imaging used.  Abbreviations as **Table 1**. | | | | | | | | | | | | |

## **Supplemental Table 4.** Outcomes according to frailty and device type in the intention-to-treat population

|  | **Acurate Neo** | | **SAPIEN 3** | | **Acurate Neo vs. SAPIEN 3** | | **Frailty vs. No frailty** | | **Interaction Hazard Ratio (Acurate Neo x Frailty)** | |
| --- | --- | --- | --- | --- | --- | --- | --- | --- | --- | --- |
|  | **Frailty**  **N = 68** | **No frailty**  **N = 304** | **Frailty**  **N = 54** | **No frailty**  **N = 313** | **Hazard or Risk ratio (95% CI)** | **P value** | **Hazard or Risk ratio (95% CI)** | **P value** | **interaction Hazard or Risk ratio (95% CI)** | **interaction P value** |
| **30-days outcomes^*^** |  |  |  |  |  |  |  |  |  |  |
| Overall mortality, n (%) | 4 (6.0%) | 5 (1.7%) | 1 (1.9%) | 2 (0.6%) | 2.60 (0.50-13.38) | 0.25 | 2.94 (0.27-32.47) | 0.38 | 1.24 (0.08-19.1) | 0.88 |
| Cardiovascular mortality, n (%) | 3 (4.5%) | 5 (1.7%) | 1 (1.9%) | 2 (0.6%) | 2.60 (0.50-13.4) | 0.25 | 2.94 (0.27-32.5) | 0.38 | 0.93 (0.06-15.2) | 0.96 |
| All stroke, n (%) | 1 (1.5%) | 6 (2.0%) | 1 (1.9%) | 10 (3.2%) | 0.62 (0.23-1.70) | 0.35 | 0.59 (0.07-4.58) | 0.61 | 1.26 (0.07-24.1) | 0.88 |
| Hospitalization^+^, n (%) | 1 (1.6%) | 3 (1.0%) | 0 (0.0%) | 5 (1.6%) | 0.62 (0.15-2.59) | 0.51 | - | - | - | - |
| **1-year outcomes^*^** |  |  |  |  |  |  |  |  |  |  |
| Clinical efficacy, n (%) | 37/58 (63.8%) | 195/260 (75.0%) | 34/45 (75.6%) | 195/270 (72.2%) | 1.04 (0.94-1.15) | 0.47 | 1.05 (0.87-1.26) | 0.63 | 0.81 (0.62-1.07) | 0.14 |
| Favorable outcome, n (%) | 36/58 (62.1%) | 198/260 (76.2%) | 31/45 (68.9%) | 200/270 (74.1%) | 1.03 (0.93-1.13) | 0.58 | 0.93 (0.75-1.15) | 0.50 | 0.88 (0.65-1.18) | 0.39 |
| Acceptable outcome, n (%) | 6/58 (10.3%) | 12/260 (4.6%) | 5/45 (11.1%) | 19/270 (7.0%) | 0.66 (0.32-1.32) | 0.24 | 1.58 (0.62-4.02) | 0.34 | 1.42 (0.38-5.34) | 0.60 |
| Unfavorable outcome, n (%) | 16/58 (27.6%) | 50/260 (19.2%) | 9/45 (20.0%) | 51/270 (18.9%) | 1.02 (0.72-1.45) | 0.92 | 1.06 (0.56-2.00) | 0.86 | 1.35 (0.61-3.01) | 0.46 |
| Overall mortality, n (%) | 11 (16.5%) | 29 (9.7%) | 6 (11.6%) | 24 (7.8%) | 1.26 (0.73-2.16) | 0.41 | 1.51 (0.62-3.69) | 0.37 | 1.19 (0.38-3.69) | 0.76 |
| Cardiovascular mortality, n (%) | 7 (10.8%) | 18 (6.1%) | 4 (7.8%) | 15 (4.9%) | 1.25 (0.63-2.47) | 0.53 | 1.60 (0.53-4.83) | 0.40 | 1.15 (0.28-4.69) | 0.85 |
| All stroke, n (%) | 3 (5.0%) | 14 (4.8%) | 1 (1.9%) | 14 (4.5%) | 1.03 (0.49-2.16) | 0.94 | 0.42 (0.06-3.19) | 0.40 | 2.36 (0.22-25.49) | 0.48 |
| Hospitalization^+^, n (%) | 8 (13.1%) | 20 (7.1%) | 7 (14.2%) | 34 (11.3%) | 0.60 (0.34-1.04) | 0.068 | 1.24 (0.55-2.81) | 0.60 | 1.58 (0.50-5.0) | 0.44 |
| **3-year outcomes^*^** |  |  |  |  |  |  |  |  |  |  |
| Clinical efficacy, n (%) | 25/57 (43.9%) | 142/240 (59.2%) | 20/41 (48.8%) | 135/253 (53.4%) | 1.11 (0.95-1.30) | 0.20 | 0.91 (0.65-1.28) | 0.60 | 0.81 (0.51-1.28) | 0.37 |
| Favorable outcome, n (%) | 23/57 (40.4%) | 150/240 (62.5%) | 20/41 (48.8%) | 148/253 (58.5%) | 1.07 (0.93-1.23) | 0.36 | 0.83 (0.60-1.16) | 0.28 | 0.77 (0.49-1.24) | 0.28 |
| Acceptable outcome, n (%) | 5/57 (8.8%) | 14/240 (5.8%) | 3/41 (7.3%) | 10/253 (4.0%) | 1.48 (0.67-3.26) | 0.34 | 1.85 (0.53-6.45) | 0.33 | 0.81 (0.17-3.97) | 0.80 |
| Unfavorable outcome, n (%) | 29/57 (50.9%) | 76/240 (31.7%) | 18/41 (43.9%) | 95/253 (37.5%) | 0.84 (0.66-1.08) | 0.17 | 1.17 (0.80-1.71) | 0.42 | 1.37 (0.84-2.25) | 0.21 |
| Overall mortality, n (%) | 21 (32.5%) | 63 (21.7%) | 13 (27.4%) | 72 (24.0%) | 0.92 (0.66-1.29) | 0.64 | 1.17 (0.65-2.11) | 0.60 | 1.37 (0.63-2.96) | 0.42 |
| Cardiovascular mortality, n (%) | 14 (23.3%) | 44 (15.8%) | 8 (17.8%) | 49 (17.1%) | 0.95 (0.63-1.42) | 0.79 | 1.06 (0.50-2.24) | 0.88 | 1.45 (0.55-3.77) | 0.45 |
| All stroke, n (%) | 4 (6.1%) | 17 (5.7%) | 2 (4.1%) | 18 (5.8%) | 0.98 (0.50-1.89) | 0.94 | 0.66 (0.15-2.84) | 0.58 | 1.58 (0.26-9.80) | 0.62 |
| Hospitalization^+^, n (%) | 12 (21.0%) | 36 (13.6%) | 10 (21.6%) | 52 (18.2%) | 0.71 (0.47-1.09) | 0.12 | 1.23 (0.62-2.41) | 0.56 | 1.39 (0.54-3.56) | 0.49 |
| Depicted are counts of events (first occurrence per patient only, % from Kaplan-Meier estimates, Wald test) for mortality, stroke and hospitalization. Clinically efficacy are counts with risk ratios and chisquare tests. Hazard ratio or Risk ratios from full-factorial models.  *N = 8 patients withdrew consent before 30 days and no clinical outcomes were recorded (n=2 frail and n=6 not frail patients). n=106 alive patients did not have KCCQ score at baseline or 1 year follow-up, so are not in the denominator; n=148 alive patients did not have KCCQ score at baseline or 3 years follow-up, so are not in the denominator.  +Hospitalization for valve-related dysfunction or heart failure.  Clinical efficacy: Freedom from all-cause mortality AND Freedom from all stroke AND Freedom from hospitalization for procedure- or valve-related causes AND Freedom from Unfavorable outcome; Favorable outcome: a patient is alive, AND has a KCCQ Overall Summary score ≥60, AND has not had a decline of >10 points in the KCCQ Overall Summary score from baseline; Acceptable outcome: [a patient is alive, AND has a KCCQ Overall Summary score ≥45, AND has not had a decline of >10 points in the KCCQ Overall Summary score from baseline; Unfavorable outcome: [a patient is not alive] OR [is alive AND has either a KCCQ Overall Summary score <45 OR has not had a decline of >10 points in the KCCQ Overall Summary score from baseline.  Abbreviations as in **Table 1-3**. | | | | | | | | | | |

## **Supplemental Table 5.** Outcomes according to frailty and device type in the per-protocol population

|  | **Acurate Neo** | | **SAPIEN 3** | | **Acurate Neo vs. SAPIEN 3** | | **Frailty vs. No frailty** | | **Interaction Hazard Ratio (Acurate Neo x Frailty)** | |
| --- | --- | --- | --- | --- | --- | --- | --- | --- | --- | --- |
|  | **Frailty**  **N = 53** | **No frailty**  **N = 315** | **Frailty**  **N = 67** | **No frailty**  **N = 297** | **Hazard or Risk ratio (95% CI)** | **P value** | **Hazard or Risk ratio (95% CI)** | **P value** | **interaction Hazard or Risk ratio (95% CI)** | **interaction P value** |
| **30-days outcomes^*^** |  |  |  |  |  |  |  |  |  |  |
| Overall mortality, n (%) | 0 | 1 (0.3%) | 3 (4.5%) | 4 (1.4%) | 0.23 (0.03-2.08) | 0.19 | 3.36 (0.75-15.0) | 0.11 | - | - |
| Cardiovascular mortality, n (%) | 0 | 1 (0.3%) | 2 (3.1%) | 4 (1.4%) | 0.23 (0.03-2.08) | 0.19 | 2.24 (0.41-12.2) | 0.35 | - | - |
| All stroke, n (%) | 1 (1.9%) | 10 (3.2%) | 1 (1.5%) | 6 (2.0%) | 1.56 (0.57-4.30) | 0.39 | 0.73 (0.09-6.09) | 0.77 | 0.82 (0.04-15.7) | 0.90 |
| Hospitalization^+^, n (%) | 0 | 5 (1.6%) | 1 (1.6%) | 3 (1.0%) | 1.56 (0.37-6.53) | 0.54 | 1.52 (0.16-14.6) | 0.72 | - | - |
| **1-year outcomes^*^** |  |  |  |  |  |  |  |  |  |  |
| Clinical efficacy, n (%) | 34/44 (77.3%) | 198/272 (72.8%) | 37/57 (64.9%) | 192/255 (75.3%) | 0.97 (0.87-1.07) | 0.51 | 0.86 (0.70-1.06) | 0.15 | 1.23 (0.94-1.61) | 0.13 |
| Favorable outcome, n (%) | 31/44 (70.5%) | 203/272 (74.6%) | 36/57 (63.2%) | 195/255 (76.5%) | 0.98 (0.89-1.08) | 0.62 | 0.83 (0.67-1.02) | 0.074 | 1.14 (0.85-1.53) | 0.37 |
| Acceptable outcome, n (%) | 5/44 (11.4%) | 19/272 (7.0%) | 6/57 (10.5%) | 12/255 (4.7%) | 1.48 (0.74-3.00) | 0.27 | 2.24 (0.88-5.71) | 0.092 | 0.73 (0.19-2.73) | 0.64 |
| Unfavorable outcome, n (%) | 8/44 (18.2%) | 50/272 (18.4%) | 15/57 (26.3%) | 48/255 (18.8%) | 0.98 (0.68-1.40) | 0.90 | 1.40 (0.84-2.31) | 0.19 | 0.71 (0.30-1.64) | 0.42 |
| Overall mortality, n (%) | 5 (10.0%) | 23 (7.4%) | 10 (15.2%) | 27 (9.3%) | 0.79 (0.45-1.38) | 0.40 | 1.72 (0.83-3.56) | 0.14 | 0.78 (0.23-2.60) | 0.68 |
| Cardiovascular mortality, n (%) | 3 (6.1%) | 14 (4.5%) | 6 (9.5%) | 16 (5.6%) | 0.81 (0.40-1.66) | 0.57 | 1.74 (0.68-4.45) | 0.25 | 0.75 (0.16-3.59) | 0.72 |
| All stroke, n (%) | 1 (1.9%) | 14 (4.5%) | 3 (5.0%) | 14 (4.9%) | 0.94 (0.45-1.97) | 0.87 | 0.97 (0.28-3.37) | 0.96 | 0.44 (0.04-4.78) | 0.50 |
| Hospitalization^+^, n (%) | 7 (14.2%) | 35 (11.5%) | 8 (13.1%) | 19 (6.9%) | 1.75 (1.00-3.06) | 0.050 | 2.02 (0.89-4.62) | 0.095 | 0.61 (0.19-1.93) | 0.40 |
| **3-year outcomes^*^** |  |  |  |  |  |  |  |  |  |  |
| Clinical efficacy, n (%) | 20/40 (50.0%) | 136/254 (53.5%) | 25/56 (44.6%) | 141/236 (59.7%) | 0.90 (0.77-1.05) | 0.17 | 0.75 (0.55-1.02) | 0.066 | 1.25 (0.79-1.97) | 0.34 |
| Favorable outcome, n (%) | 19/40 (47.5%) | 149/254 (58.7%) | 24/56 (42.9%) | 149/236 (63.1%) | 0.93 (0.81-1.07) | 0.31 | 0.68 (0.49-0.93) | 0.017 | 1.19 (0.75-1.90) | 0.46 |
| Acceptable outcome, n (%) | 4/40 (10.0%) | 10/254 (3.9%) | 4/56 (7.1%) | 14/236 (5.9%) | 0.66 (0.30-1.47) | 0.31 | 1.20 (0.41-3.52) | 0.73 | 2.11 (0.45-9.89) | 0.34 |
| Unfavorable outcome, n (%) | 17/40 (42.5%) | 95/254 (37.4%) | 28/56 (50.0%) | 73/236 (30.9%) | 1.21 (0.94-1.55) | 0.13 | 1.62 (1.17-2.24) | 0.004 | 0.70 (0.42-1.17) | 0.18 |
| Overall mortality, n (%) | 12 (26.1%) | 72 (23.7%) | 20 (31.5%) | 60 (21.2%) | 1.10 (0.78-1.55) | 0.58 | 1.58 (0.95-2.62) | 0.077 | 0.70 (0.31-1.54) | 0.37 |
| Cardiovascular mortality, n (%) | 7 (16.2%) | 49 (16.9%) | 13 (22.2%) | 41 (15.1%) | 1.10 (0.73-1.66) | 0.66 | 1.51 (0.81-2.81) | 0.20 | 0.63 (0.23-1.72) | 0.37 |
| All stroke, n (%) | 2 (4.2%) | 18 (5.8%) | 4 (6.2%) | 17 (5.9%) | 0.99 (0.51-1.92) | 0.98 | 1.04 (0.35-3.08) | 0.95 | 0.66 (0.11-4.06) | 0.65 |
| Hospitalization^+^, n (%) | 10 (21.6%) | 53 (18.3%) | 12 (21.0%) | 35 (13.5%) | 1.43 (0.93-2.19) | 0.10 | 1.72 (0.89-3.31) | 0.11 | 0.71 (0.28-1.82) | 0.47 |
| N =7 patients with no TAVI implanted were excluded.  Depicted are counts of events (first occurrence per patient only, % from Kaplan-Meier estimates, Wald test) for mortality, stroke and hospitalization. Clinically efficacy are counts with risk ratios and chisquare tests. Hazard ratio or Risk ratios from full-factorial models.  *N = 8 patients withdrew consent before 30 days and no clinical outcomes were recorded (n=2 frail and n=6 not frail patients). n=106 alive patients did not have KCCQ score at baseline or 1 year follow-up, so are not in the denominator; n=148 alive patients did not have KCCQ score at baseline or 3 years follow-up, so are not in the denominator.  +Hospitalization for valve-related dysfunction or heart failure.  Clinical efficacy: Freedom from all-cause mortality AND Freedom from all stroke AND Freedom from hospitalization for procedure- or valve-related causes AND Freedom from Unfavorable outcome; Favorable outcome: a patient is alive, AND has a KCCQ Overall Summary score ≥60, AND has not had a decline of >10 points in the KCCQ Overall Summary score from baseline; Acceptable outcome: [a patient is alive, AND has a KCCQ Overall Summary score ≥45, AND has not had a decline of >10 points in the KCCQ Overall Summary score from baseline; Unfavorable outcome: [a patient is not alive] OR [is alive AND has either a KCCQ Overall Summary score <45 OR has not had a decline of >10 points in the KCCQ Overall Summary score from baseline.  Abbreviations as in **Table 1-3**. | | | | | | | | | | |

## **Supplemental Table 6.** Baseline and procedural characteristics according to general outcome at 3 years in patients with and without frailty

|  | **Patients with frailty**  **(N = 98)** | | | | **Patients without frailty**  **(N = 493)** | | | |
| --- | --- | --- | --- | --- | --- | --- | --- | --- |
|  | **Favorable outcomes**  **N = 43** | **Acceptable outcomes**  **N = 8** | **Unfavorable outcomes**  **N = 47** | **p value** | **Favorable outcomes**  **N = 298** | **Acceptable outcomes**  **N = 24** | **Unfavorable outcomes**  **N = 171** | **p value** |
| Age, years | 82.2 ± 4.0 | 80.8 ± 4.6 | 81.9 ± 3.5 | 0.63 | 82.1 ± 3.9 | 84.0 ± 3.5 | 83.7 ± 4.6 | <0.001 |
| Female, n (%) | 28 (65%) | 4 (50%) | 34 (72%) | 0.42 | 176 (59%) | 14 (58%) | 79 (46%) | 0.025 |
| Body mass index, kg/cm² | 27.9 ± 4.6 | 26.8 ± 3.4 | 28.2 ± 5.1 | 0.76 | 27.7 ± 4.3 | 28.4 ± 4.7 | 27.3 ± 4.7 | 0.46 |
| STS-PROM, % | 4.3 ± 3.0 | 2.9 ± 0.6 | 5.6 ± 5.8 | 0.18 | 3.6 ± 1.8 | 5.1 ± 3.0 | 5.2 ± 3.5 | <0.001 |
| NYHA III or IV, n (%) | 37 (86%) | 8 (100%) | 42 (89%) | 0.51 | 203 (68%) | 22 (92%) | 133 (78%) | 0.008 |
| CCS grade III or IV, n (%) | 4 (9%) | 0 | 2 (4%) | 0.46 | 16 (5%) | 5 (21%) | 7 (4%) | 0.004 |
| Syncope, (%) | 10 (23%) | 0 | 1 (2%) | 0.004 | 35 (12%) | 4 (17%) | 16 (9%) | 0.50 |
| Low activity, n (%) | 24 (56%) | 4 (50%) | 23 (55%) | 0.96 | 82 (28%) | 15 (65%) | 73 (47%) | <0.001 |
| Dementia/cognitive impairment, n (%) | 1 (2%) | 0 | 0 | 0.52 | 3 (1%) | 1 (4%) | 4 (2%) | 0.33 |
| Serum albumin <35 g/L, n (%) | 5 (14%) | 0 | 8 (23%) | 0.29 | 47 (22%) | 4 (24%) | 42 (33%) | 0.075 |
| Serum albumin, g/L | 41.8 ± 5.8 | 40.8 ± 3.2 | 38.5 ± 4.8 | 0.034 | 39.2 ± 5.1 | 39.3 ± 5.3 | 37.0 ± 6.2 | 0.002 |
| **Comorbidities** |  |  |  |  |  |  |  |  |
| Hypertension, n (%) | 41 (95%) | 6 (75%) | 44 (94%) | 0.12 | 274 (92%) | 22 (92%) | 152 (89%) | 0.54 |
| Diabetes mellitus, n (%) | 7 (16%) | 3 (38%) | 20 (43%) | 0.024 | 85 (29%) | 6 (25%) | 61 (36%) | 0.22 |
| Creatinine concentration >2 mg/dL, n (%) | 0 | 1 (12%) | 3 (6%) | 0.14 | 7 (2%) | 0 | 11 (6%) | 0.047 |
| Coronary artery disease, n (%) | 23 (53%) | 5 (62%) | 24 (51%) | 0.83 | 180 (60%) | 18 (75%) | 101 (59%) | 0.32 |
| Previous myocardial infarction, n (%) | 6 (14%) | 0 | 8 (17%) | 0.44 | 35 (12%) | 4 (17%) | 19 (11%) | 0.73 |
| Previous stroke or TIA, n (%) | 6 (14%) | 2 (25%) | 10 (21%) | 0.59 | 23 (8%) | 4 (17%) | 27 (16%) | 0.017 |
| COPD, n (%) | 6 (14%) | 1 (12%) | 7 (15%) | 0.98 | 21 (7%) | 2 (8%) | 28 (16%) | 0.006 |
| Atrial fibrillation, n (%) | 11 (26%) | 3 (38%) | 25 (53%) | 0.028 | 84 (28%) | 10 (42%) | 86 (50%) | <0.001 |
| Extracardiac arteriopathy, n (%) | 5 (12%) | 0 | 4 (9%) | 0.57 | 36 (12%) | 4 (17%) | 16 (9%) | 0.47 |
| **Echocardiography*** |  |  |  |  |  |  |  |  |
| Aortic valve area, cm² | 0.69 ± 0.20 | 0.67 ± 0.16 | 0.71 ± 0.18 | 0.74 | 0.73 ± 0.19 | 0.71 ± 0.17 | 0.73 ± 0.19 | 0.88 |
| Mean aortic valve gradient, mmHg | 47.9 ± 19.4 | 45.9 ± 9.8 | 40.0 ± 18.0 | 0.12 | 43.4 ± 15.4 | 36.3 ± 17.9 | 40.3 ± 17.2 | 0.028 |
| Left ventricular ejection fraction, % | 54.9 ± 13.4 | 60.1 ± 5.6 | 55.2 ± 9.8 | 0.47 | 57.7 ± 10.8 | 54.4 ± 10.2 | 56.3 ± 11.3 | 0.19 |
| Moderate or severe aortic regurgitation, n (%) | 3 (8%) | 3 (43%) | 8 (21%) | 0.044 | 29 (10%) | 5 (22%) | 14 (9%) | 0.16 |
| Moderate or severe mitral regurgitation, n (%) | 8 (19%) | 2 (25%) | 4 (9%) | 0.26 | 36 (12%) | 7 (29%) | 33 (20%) | 0.017 |
| Moderate or severe tricuspid regurgitation, n (%) | 4 (9%) | 0 | 6 (13%) | 0.50 | 24 (8%) | 3 (14%) | 28 (17%) | 0.024 |
| **Procedural characteristics** |  |  |  |  |  |  |  |  |
| Transfemoral TAVR not initiated, n (%) | 0 | 0 | 2 (4%) | 0.33 | 0 | 0 | 3 (2%) | 0.058 |
| Transfemoral access mode, n (%) |  |  |  |  |  |  |  | 0.021 |
| percutaneous | 43 (100%) | 8 (100%) | 45 (100%) |  | 298 (100%) | 23 (96%) | 166 (99%) | 0.021 |
| surgical cut-down | 0 | 0 | 0 |  | 0 | 1 (4%) | 2 (1%) | 0.021 |
| General anesthesia, n (%) | 6 (14%) | 0 | 6 (13%) | 0.53 | 67 (22%) | 5 (21%) | 42 (25%) | 0.79 |
| Valve type implanted, n (%) |  |  |  | 0.73 |  |  |  | 0.24 |
| SAPIEN 3 | 19 (44%) | 4 (50%) | 17 (38%) | 0.73 | 149 (50%) | 10 (42%) | 95 (57%) | 0.24 |
| Acurate Neo | 24 (56%) | 4 (50%) | 28 (62%) | 0.73 | 149 (50%) | 14 (58%) | 73 (43%) | 0.24 |
| Valve size implanted, mm | 25.1 ± 1.5 | 25.6 ± 1.3 | 25.3 ± 1.6 | 0.56 | 25.2 ± 1.7 | 25.1 ± 1.5 | 25.2 ± 1.6 | 0.99 |
| Moderate or severe paravalvular regurgitation post-procedure, n (%) | 1 (2%) | 0 | 2 (5%) | 0.71 | 10 (3%) | 0 | 8 (5%) | 0.42 |
| **Procedural complications within 30 days** |  |  |  |  |  |  |  |  |
| Valve malposition | 0 | 0 | 1 (2%) | 0.58 | 4 (1%) | 0 | 1 (1%) | 0.64 |
| Coronary artery obstruction due to TAVI | 0 | 0 | 0 |  | 0 | 0 | 1 (1%) | 0.39 |
| Myocardial infarction | 0 | 0 | 0 |  | 1 | 0 | 2 (1%) | 0.50 |
| Implantation of multiple valves | 1 (2%) | 0 | 1 (2%) | 0.91 | 6 (2%) | 0 | 2 (1%) | 0.65 |
| Cardiac tamponade | 1 (2%) | 0 | 0 | 0.52 | 1 | 0 | 5 (3%) | 0.041 |
| Annular rupture | 0 | 0 | 0 |  | 0 | 0 | 1 (1%) | 0.39 |
| Left ventricular perforation | 0 | 0 | 0 |  | 0 | 0 | 1 (1%) | 0.39 |
| Conversion to open heart surgery | 0 | 0 | 0 |  | 1 | 0 | 2 (1%) | 0.50 |
| Immediate procedural death | 0 | 0 | 1 (2%) | 0.58 | 0 | 0 | 3 (2%) | 0.058 |
| Depicted are counts (% with chisquare test p-values) or means with standard deviations (with ANOVA F-test p-values).  *n=8 patients withdrew consent before 30 days and no clinical outcomes were recorded (n=2 frail and n=6 not frail patients). n=148 alive patients did not have KCCQ score at baseline or 3 years follow-up, so are not in the denominator. | | | | | | | | |
